# Development of a User-Informed Decision Aid for Contraceptive Decision-Making Among Adolescents and Young Adults: Qualitative Study

**DOI:** 10.2196/82306

**Published:** 2026-07-21

**Authors:** Sophie Allende-Richter, Susan Gonzalez, Shravya Sathi, Alix Quinn, Madeline Pereira, Brett Nava-Coulter, Anna Revette, Christopher Landrigan, Karen Sepucha

**Affiliations:** 1 Division of General Pediatrics Department of Pediatrics Boston Children's Hospital Boston, MA United States; 2 Harvard Medical School Boston, MA United States; 3 College of Science Northeastern University Boston, MA United States; 4 Fenway Health Boston, MA United States; 5 Dana Farber Cancer Institute Boston, MA United States; 6 Health Decision Sciences Center Massachusetts General Hospital Boston, MA United States

**Keywords:** adolescents and young adults, female individuals, shared decision-making, decision aid, patient preferences, contraceptive method indications, noncontraceptive indications, youth-informed decision aid development

## Abstract

**Background:**

Adolescents and young adults seeking contraceptive care face many considerations related to differences in contraceptive indications and knowledge, which are often overlooked and can lead to contraceptive nonadherence or nonuse as well as adverse health outcomes.

**Objective:**

This study aimed to develop a digital decision aid that meets the contraceptive decisional needs of adolescents and young adults from diverse backgrounds.

**Methods:**

We developed a web-based decision aid using a user-centered design framework and the International Patient Decision Aid Standards. The design and development process was informed by a literature review and consultations with scientific experts, health informatics specialists, clinicians experienced in adolescent reproductive health, and a diverse group of adolescent and young adult advisors. We gathered feedback on the decision aid’s content and functionality from clinicians, adolescents, and young adult stakeholders during focus group interviews conducted across 2 user testing cycles. Each focus group was recorded and transcribed, and the data were analyzed using a focus group guide to identify key attributes, patterns, and perspectives among users. Two qualitative researchers used rapid qualitative analysis to explore and summarize findings across 4 key domains, which contributed to the refinement of the decision aid content and the improvement of its functionality.

**Results:**

Twenty-four clinicians, adolescents, and young adult participants from diverse backgrounds shared their perspectives on the decision aid’s content, relevance, design, and usability across 2 user testing cycles conducted from February 2023 to June 2024. The decision aid includes a survey, a decision algorithm that generates a summary of contraceptive method recommendations, infographics, and a health care provider summary view. The decision algorithm applies a weighted scoring system ranging from +1 or −1 to +10 or −10 for each method, reflecting the user’s primary indication for seeking contraception, contraceptive use preferences, and relevant health history.

**Conclusions:**

This approach allowed us to capture rich perspectives from a diverse group of stakeholders that accounted for the unique contraceptive decision-making needs of adolescents and young adults, resulting in a functional, youth-informed decision aid prototype, *MyPlanMyChoice*, ready for pilot and feasibility evaluation in a clinical setting.

## Introduction

Contraceptive methods (CMs) vary widely in formulations and indications (ie, menstrual cycle regulation) beyond pregnancy prevention [1-3]. Adolescents and young adults (AYAs) seeking contraceptives face numerous considerations (eg, weight gain and pain with long-acting CM insertion) that are often overlooked by health care providers due to competing priorities and a lack of adequate contraceptive information, which can lead to CM misuse or nonuse and undesirable outcomes. AYAs also often rely on social media for contraceptive information [4-8]. AYAs from racial and ethnic minority groups are more likely to report mistrust in contraceptive use and to forego or discontinue a method [4]. Furthermore, AYAs with a nonconforming sexual orientation, gender identity or form of expression (SOGIE) are less likely to receive contraceptive counseling that meets their needs outside of a gender-affirming care clinic (eg, counseling regarding menstrual suppression) [9,10]. Therefore, developing a culturally and developmentally tailored intervention to support AYAs’ unique contraceptive indications and decisional needs is critical.

The American Academy of Pediatrics recommends a patient-centered approach to contraceptive counseling to improve method uptake and prevent early discontinuation and adverse outcomes [1]. Shared decision-making (SDM)—the process of involving patients in health decisions about treatment options, benefits, and harms—enables patients to make evidence-informed choices that align with what matters most to them [11,12]. This approach reduces decisional conflict and enhances patients’ knowledge, treatment adherence, and health outcomes [11-13]. SDM is considered the pinnacle of patient-centered care and is standard practice for adult women’s contraceptive counseling [11,14,15]. Although several contraceptive decision support tools have been shown to improve contraceptive knowledge among AYAs, their effectiveness in supporting AYA contraceptive choice, methods use, and treatment satisfaction remains unclear [16,17]. Furthermore, few of these tools meet the patient decision aid (DA) standards set by the International Patient Decision Aid Standards (IPDAS), and many have not been designed to address the unique needs of a diverse AYA population, including individuals who identify as nonbinary [17,18]. Finally, few have been rigorously evaluated, tested in diverse AYA populations, or successfully implemented in clinical practice following clinical trials (for a summary of existing contraceptive decision support tools, refer to Multimedia Appendix 1) [16-29]. To address these gaps, we aimed to develop a web-based DA that addresses the unique contraceptive decisional needs of AYAs from diverse backgrounds, following an evidence-based framework for web-based DA for patients and the IPDAS [18,30].

## Methods

### Systemic Development Process Framework

**Figure 1:**
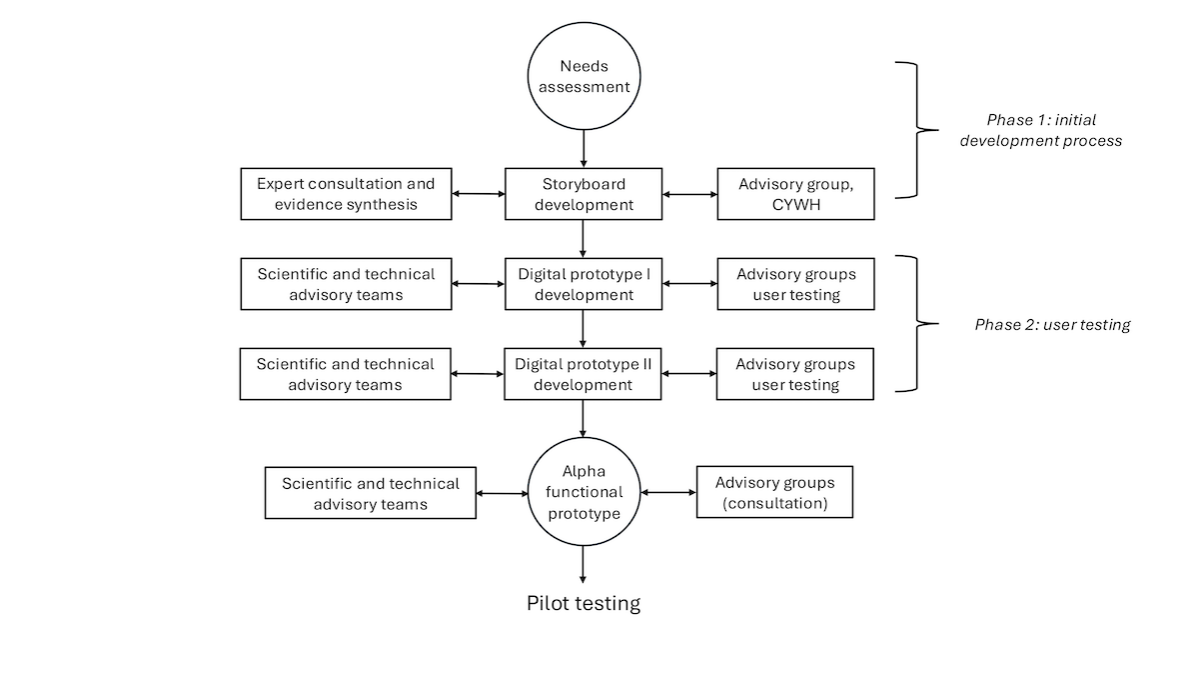
Decision aid development process and stages. Phase 1 consisted of the needs assessment and storyboard development. Phase 2 consisted of user testing, resulting in an alpha functional prototype. CYWH: Center for Young Women’s Health.

We drew upon the Elwyn et al [30] framework for web-based patient DAs to develop our DA, which involved two main phases: (1) initial development and (2) user testing (Figure 1). These phases were coordinated by the research team in collaboration with (1) an advisory group of stakeholder representatives, including a youth advisory group, clinicians, and community partners, to provide high-level guidance; (2) scientific experts with content knowledge in areas such as DAs and adolescent contraception; (3) a technical production team, including hospital health informatics experts and software consultants; and (4) clinicians and patient stakeholders, who tested the app as prospective users. Each group contributed its expertise and perspective to the research team at key points in the DA development process (Figure 1). However, these groups did not have editing privileges for the DA content [30].

### Phase 1: Initial Development Process

#### Content Specification

This phase consisted of (1) a needs assessment conducted in our clinic to evaluate CM use, as well as patients’ and health care providers’ perspectives on reproductive health services; and (2) an evidence synthesis based on the Centers for Disease Control and Prevention Contraceptive Clinical Guidelines, the American Academy of Pediatrics, and a literature review [1,31,32]. We consulted experts in patient DA development, including internationally recognized decision scientists, health informatics, and clinicians specialized in adolescents reproductive health, as well as youth advisors from the Center for Young Women’s Health Patient Advisory Group throughout the DA development process. This process resulted in the creation of a storyboard, which served as the basis for our DA structure, content, and illustrations (Figure 1).

#### Design Phase

This phase was guided by the IPDAS and value clarification methods to elicit patients’ contraceptive preferences based on each method’s specific attributes that align with their values and what matters most to them (ie, pregnancy intent and timing and menstrual suppression) [18]. Throughout this phase, we worked closely with the technical production and advisory groups, as well as DA experts. This process resulted in our first DA prototype, *MyPlanMyChoice*, compatible with iOS and Android mobile devices (Figure 1; Table 1).

**Table 1:** Correspondence between the International Patient Decision Aid Standards (IPDAS) criterion and the structure and content of MyPlanMyChoice.

IPDAS criterion		DA^a^
Development process		
	Present information in a balanced manner	The DA presents attributes for CMs^b^ in addition to unwanted pregnancy prevention and allows the user to identify which factors matter to them. The summary page includes pros and cons for all methods, which users can access at any time while using the DA.
	Has a systematic development process	We drew upon Elwyn et al [30] user-centered design framework for DA development [18,30]. See Methods section and Figure 1.
	Use updated scientific evidence that is cited in a reference section or technical document	See Methods section. Landing page informs user that the DA was developed using the American Academy of Pediatrics, American College of Gynecology and Obstetrics, and Center for Disease Control US Selected Practice Recommendations for Contraceptive use and Medical Eligibility Criteria for Contraceptive Use guidelines [1,31,32].
	Disclose conflicts of interest	There are no conflicts of interest to disclose.
	Uses plain language	Based on user testing feedback, reading level was adjusted to improve content clarity (eg, “stop or decrease periods” rather than “menstrual suppression”) and the language was updated to be non-gendered (eg, “pregnant person” rather than “pregnant woman,” and “external condom” rather than “male condom”).
Content		
	Provide information about options in sufficient detail for decision making	CM summary screen: presents CM options with a short description of the methods’ mode of action, illustration, and a patient-friendly “pros and cons” list that includes CM effectiveness: pregnancy prevention rate, percentage of users with menstrual suppression, side effects, and additional method-specific noncontraceptive benefits. The addition of a navigation button allows user to access the CM summary page at any time while using the DA.
	Present probabilities of outcomes in an unbiased and understandable way	CM summary screen: includes statistics for CM effectiveness: pregnancy prevention rate, percentage of users with menstrual suppression, side effects, and additional method-specific noncontraceptive benefits.
	Include methods for clarifying and expressing patients’ values	CM indication: the user indicates their primary reason for seeking CM among several indications (pregnancy prevention, menstrual suppression, etc) on a visual analog scale. Preference elicitation: user selects the least and most preferred CM attributes on a best-worst scale. This process uses tradeoffs consideration (eg, weight gain and privacy consideration) to allow user to identify preferred CM [12,18].
	Include structured guidance in deliberation and communication	Introduction screen: welcomes user to the DA, describes the purpose of the DA and emphasizes discussion with health care provider. CM recommendation page: provides user with top methods recommended based on the user’s responses and prompts user to engage in a conversation with their health care provider prior to making a final choice.

^a^DA: decision aid.

^b^CM: contraceptive method.

The DA’s architectural structure has two components: (1) a Flutter app (Berkeley Software Distribution 3–Clause license) that allows deployment for both iOS and Android devices, hosted on TestFlight (Apple Inc) accounts and not available to the public, and (2) a Rails (Massachusetts Institute of Technology license) back end that provides an application programming interface for the Flutter app, hosted on a Heroku server (Salesforce Inc) and enable user data to be transferred to Research Electronic Data Capture (REDCap; Vanderbilt University) software for data collection (DA architecture map available in Multimedia Appendix 2) [33]. The DA user interface consists of a self-administered survey and a DA described below.

**Figure 2:**
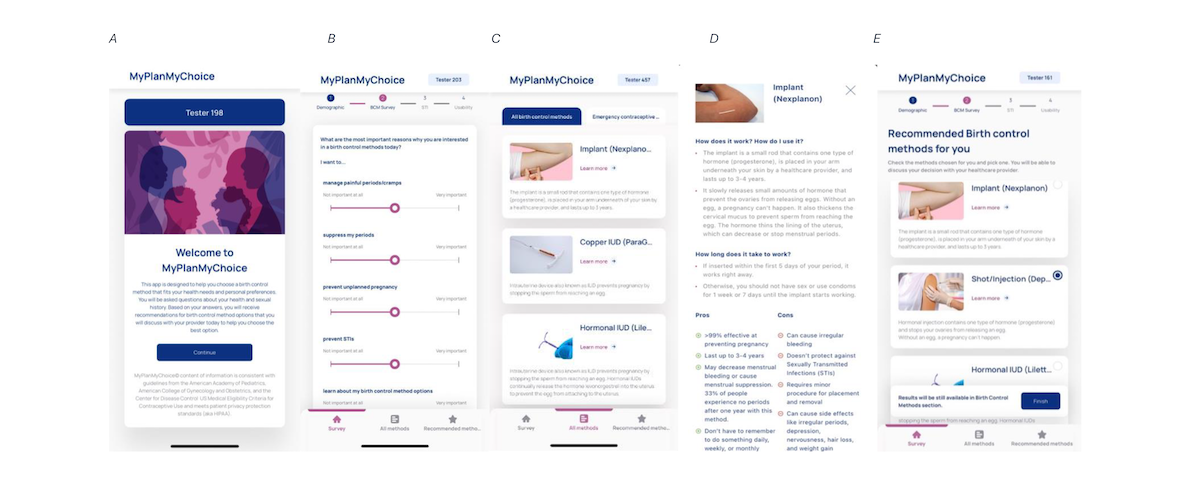
MyPlanMyChoice decision aid key content illustration. (A) The landing page introduces the purpose of the decision aid, (B) contraceptive method preference selection, (C) view of all contraceptive method options, (D) contraception method–specific information, and (E) recommended contraceptive method options.

The DA includes a self-administered survey that collects information on CM preferences and health history, educational infographics, and a built-in decision algorithm that generates personalized CM recommendations based on users’ preferences and health history. Users indicated their most and least preferred contraceptive attributes (ie, menstrual bleeding pattern and concerns about specific side effects) on a best-worst survey and reported their interest in using a specific CM on a 4-point Likert scale. The decision algorithm applied a weighted scoring system ranging from +1 or −1 to +10 or −10 to each CM, based on the user’s stated preferences, medical eligibility, and the degree to which the method’s pharmacological properties were more strongly associated with achieving the user’s desired outcome. For instance, if a user selected menstrual suppression as a preferred attribute, a score of +1 would be assigned to the injectable hormonal method and the subdermal implant, +2 to the levonorgestrel-releasing intrauterine device, and −1 to combined CMs, reflecting their relative likelihood of achieving menstrual suppression. Methods that were medically contraindicated based on the user’s health history or that the user indicated they would never consider for personal reasons received a score of −10 (or multiples thereof) to ensure that they were excluded from the recommended CM options. Conversely, methods that users indicated they were strongly interested in using or using again received a score of +10. Selection of CM attributes was informed by the US Selected Practice Recommendations for Contraceptive Use and the Centers for Disease Control and Prevention’s US Medical Eligibility Criteria for Contraceptive Use [31,32]. The decision algorithm subsequently generates a recommended CM summary of the 5 methods that best align with the user’s stated preferences and clinical profile, from which the user can select a preferred option (Figure 2; a higher-resolution version is available in Multimedia Appendix 3). Prior to implementation, the research team and scientific advisory team independently reviewed the decision algorithm scoring rules. The research team pilot-tested the algorithm in 2 iterative rounds of user testing and reviewed and addressed discrepancies between participants’ preferences and the algorithm-generated recommendations. The scientific advisory team proofread the final version before it was incorporated into the final algorithm (Figure 1).

Finally, the DA features a health care provider summary view, allowing health care providers to review the user’s primary indication for a CM; preferences regarding CM use (ie, menstrual cycle pattern or management of dysmenorrhea); and relevant health history (ie, smoking status and blood pressure [BP]), including any contraindications (ie, blood clot disorders), to discuss options and help user select a preferred CM (health care provider summary view available in Multimedia Appendix 4).

### Phase 2: User Testing

#### Overview

This phase consisted of 2 iterative rounds of testing and prototype refinement. Potential end users tested the digital prototype on their smartphones and provided feedback during semistructured focus groups to assess functionality and ensure that the *MyPlanMyChoice* content met AYAs’ contraceptive decision-making needs (Figure 1). Discrepancies between user testing responses and the decision algorithm’s recommendations were reviewed and adjudicated by the research and scientific advisory teams following each round of user testing, and the software development team implemented corresponding changes to the software (Figure 1).

#### Participant Recruitment

Participants were recruited through purposive sampling at a primary care clinic affiliated with a teaching hospital in Boston or via email directed to youth-focused community health agencies and the Harvard SOGIE Health Equity Research Collaborative. Each user testing cycle involved three distinct groups of 2 to 5 participants: (1) clinicians with expertise in AYA reproductive health, (2) cisgender female AYAs, and (3) AYA-assigned female at birth (AFAB) with nonconforming SOGIE. Each cycle of user testing included new participants to ensure a diverse range of participant perspectives in our DA development process.

#### User-Informed Design

We developed a focus group guide in collaboration with 2 qualitative researchers (AR and BN-C) to assess the following DA domains: (1) clarity of information, (2) relevance, (3) design, and (4) usability. All participants tested the DA on their smartphones—no personal information was saved—and provided feedback during 1-hour semistructured online video focus group interviews. We carried out 2 rounds of user testing, which resulted in an alpha functional prototype.

#### Analysis

This section focuses only on the qualitative analysis of the user testing phase that led to the refinement of our DA. The results of the needs assessment that informed our DA development are available in a separate publication [4]. Each focus group was recorded and transcribed, and the data were analyzed to identify key attributes, patterns, and perspectives among DA users based on the focus group guide. Two qualitative researchers used rapid qualitative analysis to explore and summarize findings across the 4 key domains. Each transcript was summarized in a structured table organized by prefigured domains associated with clarity of information, relevance, design, and usability, with additional open cells to capture any salient emergent domains. Each summary table was reviewed and discussed by at least 2 members of the interdisciplinary team to ensure consistent application of the domains and to confirm the comprehensiveness of the data synthesis, with a key focus on divergent cases and the potential for alternative interpretations to inform refinements and enhancements of the DA (Figure 1). This approach allowed more nuanced insights by incorporating diverse participants’ perspectives, rather than seeking consensus through thematic analysis [34]. The analyses were supported by NVivo (Lumivero) software.

### Ethical Considerations

The DA met hospital cybersecurity and HIPAA (Health Insurance Portability and Accountability Act) compliance. The Boston Children’s Hospital Institutional Review Board approved this study (IRB-P00042301) through expedited review procedures in accordance with Federal and US Department of Health and Human Services regulations governing the inclusion of children in research. Risks were determined to be minimal, and there was no potential for direct benefit. Parental consent for minor participants was waived, and verbal consent was obtained from all study participants. Each participant received a US $60 gift card.

## Results

Twenty-four participants, including clinicians (n=10), cisgender female AYAs (n=9), and AYA-AFABs with nonconforming SOGIE (n=5), aged 16 to 24 years, most of whom identified as White, Asian, Hispanic or Latino, or Black, contributed to our DA through 2 user testing cycles from February 2023 to June 2024 (Table 2). User testing cycle 1 included 3 focus groups comprising health care providers (n=5), cisgender female AYAs (n=4), and AFABs with nonconforming SOGIE (n=3). User testing cycle 2 involved 4 focus groups, comprising 2 health care provider groups: group 1 (n=3) and group 2 (n=2), plus 1 group of cisgender female AYAs (n=5) and 1 group of AYA-AFABs with nonconforming SOGIE (n=2). Participants’ characteristics are summarized in Table 2.

**Table 2:** Characteristics of user-testing key informants.

Participants characteristics			AYA^a^-AFAB^b^ (n=14)		Health care providers (n=10)
Age (years), median (IQR)			19 (16-25)		N/A^c^
Race and ethnicity, n (%)					
	Asian or Pacific Islander	4 (29)		1 (10)	
	Black or African American	2 (14)		1 (10)	
	Hispanic or Latino	3 (21)		1 (10)	
	Other or mixed	1 (7)		0 (0)	
	White	4 (29)		7 (70)	
Gender identity, n (%)					
	Nonbinary AYA-AFAB	5 (36)		0 (0)	
	Cis-gender female AYA	9 (64)		10 (100)	
Primary language, n (%)					
	English	13 (93)		N/A	
	Other	1 (7)		N/A	
Education level, n (%)					
	High school	6 (43)		N/A	
	Some college	5 (36)		N/A	
	Bachelor’s degree	2 (14)		N/A	
	Master’s degree	1 (7)		N/A	
Credentials, n (%)					
	Doctor of Medicine or Doctor of Osteopathic Medicine	N/A		4 (40)	
	Nurse practitioner	N/A		4 (40)	
	Other	N/A		2 (10)	
Years in specialty training, n (%)					
	1-10	N/A		5 (50)	
	11-20	N/A		4 (40)	
	21-30	N/A		1 (10)	
Experience providing reproductive health counseling, n (%)					
	Yes	N/A		10 (100)	
	No	N/A		0 (0)	
Experience treating or counseling nonbinary youth, n (%)					
	Yes	N/A		10 (100)	
	No	N/A		0 (0)	

^a^AYA: adolescent and young adult.

^b^AFAB: assigned female at birth.

^c^N/A: not available.

Key findings from the user testing analysis prompted the following changes between our first and second digital prototypes, available in Multimedia Appendix 5: (1) content refinement through the use of nongendered language throughout the app; (2) reduced emphasis on heteronormative indications for contraceptives; and (3) the inclusion of medical contraindications in the decision algorithm, along with relevant patient facing cardiometabolic screenings (BMI, BP, and smoking status), according to the US Medical Eligibility Criteria for Contraceptive Use [31,32]. Other changes included shortening the DA content to include only information that health care providers and youth participants considered most relevant and improving the end user experience by adding navigation buttons. Clinicians found the DA to be comprehensive because it addressed all the important aspects they wanted to discuss during an encounter and felt that it would save time and effectively support SDM between patients and health care providers during a visit if the health care provider summary view interfaced with the electronic health record (Multimedia Appendix 5). This iterative process improved the clarity and relevance of the DA’s information, design, and usability, resulting in a functional prototype, *MyPlanMyChoice* (Figure 2).

## Discussion

This study describes the development process of the DA, *MyPlanMyChoice,* designed to address the unique contraceptive and noncontraceptive indications of AYA-AFABs. This approach enabled us to incorporate the perspectives of a diverse group of stakeholders, including AYA-AFABs with a nonconfirming SOGIE, racial and ethnic minority groups, and adolescent health care providers, to guide the development and refinement of the DA content. This approach enabled us to prioritize aspects crucial to AYAs’ contraceptive choice, including but not limited to using inclusive language and illustrations throughout the DA, as well as presenting concerns about contraceptive side effects (ie, weight gain and unscheduled bleeding), alongside contraceptive and noncontraceptive indications (Figure 2; Multimedia Appendix 3), which health care providers may view as less critical than pregnancy prevention. However, undisclosed side effects or benefits can lead to contraceptive misinformation, lack of trust, failure to choose a CM, method dissatisfaction, and early discontinuation of CMs [4,5]. Additionally, our user testing led to the inclusion of a frequently asked questions tab to address common concerns and misconceptions (eg, infertility) and the lack of reliable sources of information, which often leads AYAs or the parents of AYAs to rely on social media as a source of information [6-8]. Furthermore, this process addressed health care providers’ perspectives on the implementation of the DA, resulting in an improved health care provider summary view that incorporates the patient’s medical history and relevant cardiometabolic risk factors, such as BMI, BP, and smoking, as well as the importance of integrating the health care provider summary view into the electronic health record in future versions of our DA to facilitate SDM during a medical encounter. Our multistage collaboration with expert advisors ensured that the proposed changes from user testing were supported by clinical evidence. Finally, the iterative nature of our testing process allowed us to refine the content, enhancing the functionality and fidelity of our DA prototype before conducting pilot testing in clinical settings.

Although we aimed to include diverse viewpoints in the development of our DA, we acknowledge several factors that limited our ability to achieve adequate representation among our user testing participants. First, we had a slightly lower proportion of AYA participants from racial and ethnic minority groups who self-identified as Black (n=2) or Hispanic or Latino (n=3) than those who identified as White (n=4) or Asian (n=4). Additionally, our sample size was relatively small, and the age distribution was skewed toward older AYAs with a median age of 19 (16-25) years (Table 2). Finally, our DA prototype was available only in English, which limited the enrollment of non–English-speaking participants to test the app and provide feedback. Nevertheless, to address these limitations, we plan to partner with AYAs from our hospital youth advisory group to inform our recruitment strategy for the next phases of our DA evaluation.

In summary, SDM is regarded as the gold standard of patient-centered care and is standard practice for adult women’s contraceptive counseling [11-15]. Although several available contraceptive decision support tools have been shown to improve contraceptive knowledge among AYAs, most have focused on pregnancy prevention, even though contraceptives have a wide range of indications. Few have involved the perspectives of AYAs from diverse SOGIE, racial, and ethnic backgrounds and their decisional needs in their design; undergone rigorous evaluation; or addressed implementation strategies. Therefore, their use in clinical settings beyond pilot testing and their effectiveness in supporting the AYAs remain unclear [17,29]. This study outlines a patient-centered approach to developing a DA that addresses the contraceptive decisional needs of AYA-AFABs from diverse racial, sexual orientation, and gender identity backgrounds. This process resulted in the development of a functional DA, *MyPlanMyChoice*, that presents a comprehensive range of CM options, including noncontraceptive indications and method side effects. This enables progress toward pilot and feasibility testing [35]. In the next phase of our study, we plan to assess the acceptability and feasibility of our DA intervention among AYAs and health care providers in a clinical setting and to explore its preliminary efficacy beyond pregnancy prevention, such as improving cardiometabolic awareness and informing on contraceptive choice. To accomplish this, we plan to administer evidence-based acceptability and decision quality scales and contraceptive knowledge preinterventional and postinterventional surveys. Additionally, we plan to follow the Consolidated Framework for Implementation Research to identify barriers and facilitators among patients and health care providers through semistructured interviews, thereby informing the development of implementation strategies and a future hybrid effectiveness-implementation trial [36].

## Data Availability

The data are provided in the Results section of the main manuscript. Additional data, including the focus group guide, are available upon request.

## Appendix

Multimedia Appendix 1 Comparative summary table of existing contraceptive decision aids.

Multimedia Appendix 2 MyPlanMyChoice architecture map.

Multimedia Appendix 3 MyPlanMyChoice user interface key content illustration.

Multimedia Appendix 4 Screenshot of the MyPlanMyChoice health care provider summary view.

Multimedia Appendix 5 Illustrative stakeholder quotes and corresponding changes implemented in MyPlanMyChoice following user testing.

## Abbreviations

AFAB: assigned female at birth
AYA: adolescent and young adult
BP: blood pressure
CM: contraceptive method
DA: decision aid
HIPAA: Health Insurance Portability and Accountability Act
IPDAS: International Patient Decision Aid Standards
REDCap: Research Electronic Data Capture
SDM: shared decision-making
SOGIE: sexual orientation, gender identity, and expression
