# Supplementary material for: Development of a User-Informed Decision Aid for Contraceptive Decision-Making Among Adolescents and Young Adults: Qualitative Study
Source: JMIR Form Res. 2026 Jul 21;10:e82306. doi: 10.2196/82306 (PMC13439044; doi:10.2196/82306)
Supplement: Supplementary file 1 [file formative_v10i1e82306_app1.docx]

| **Resource name** | **Description** | **Target population** | **Youth under 18 involved in design process?** | **Evaluation study design, setting (N= sample size, gender, age range)** | **IPDAS Content Criteria A. Provides information about options in sufficient detail for decision making** | **IPDAS Content Criteria B. Presents probabilities of outcomes in an unbiased and understandable way** | **IPDAS Content Criteria C. Includes methods for clarifying and expressing patients’ values** | **IPDAS Content Criteria D. Includes structured guidance in deliberation and communication** |
| --- | --- | --- | --- | --- | --- | --- | --- | --- |
| MyPlanMyChoice© | Tablet-based shared decision-making aid that provides recommended CMs based on user’s preferences and values | AYA AFAB of diverse racial background and SOGIE (13-25 years old) | Yes | In progress | **Yes.** Allows user to access all CM information at any time while using the application | **Yes.** Presents information about all CM options, including statistics, in patient-friendly “pros and cons” format | **Yes.** Includes visual analog scale for value clarification | **Yes.** Prompts user to engage with provider and gives provider a summary of the user’s responses to guide clinical encounter |
| [World Health Organization decision-making tool for family planning](https://iris.who.int/bitstream/handle/10665/43225/9241593229_eng.pdf?sequence=2) | Flip-chart guide for provider and patient family planning visit | All people internationally | Unknown | [Kim 2005](https://www.guttmacher.org/sites/default/files/pdfs/pubs/journals/3116205.pdf), pre-post study, clinical setting, Mexico (N=83 women ages 17-50)  [Kim 2007](https://www.sciencedirect.com/science/article/abs/pii/S0738399106004265?via%3Dihub), pre-post study, clinical setting, Nicaragua (N=421 women and 5 unknown, ages 17-50) | **Somewhat.** Includes information about all CM options but requires provider to guide patient through this information | **Somewhat.** Provides general summary of effectiveness with more information available to the provider | **Somewhat.** Asks open-ended questions to patient (i.e., “what matters to you?”) Does not explicitly elicit patient values | **Yes.** Provides guidance to provider for discussion with patient |
| [World Health Organization Medical Eligibility Criteria mobile application](https://www.who.int/news/item/29-08-2019-new-app-for-who-s-medical-eligibility-criteria-for-contraceptive-use) | Mobile app with up-to-date medical eligibility criteria, designed for medical providers to see CM recommendations based on medical conditions and patient preferences | All people internationally | Unknown | None | **Somewhat.** Includes information about all CM options but requires provider to guide patient through this information | **Somewhat.** Provides information about CM options but requires provider to guide patient through information | **Yes.** Provides recommended methods based on the patient’s selected preferences | **Yes.** Provides guidance to provider for use with patient |
| [Bedsider.org “Explore birth control options” page](https://www.bedsider.org/birth-control) | Interactive, informational website about contraceptive method options | Women aged 18-29 | No | [Antonishak 2015](https://journals.sagepub.com/doi/10.1177/1524500414566698), RCT, non-clinical setting (N=2,284 women ages 18-29) | **Yes.** Summarizes all CM options | **Yes.** Provides pregnancy prevention probabilities, summarizes side effects | **No.** Allows user to filter methods based on different characteristics but does not elicit patient values | **No.** Provides resources for user to find a healthcare provider, but no resources for discussion |
| [Planned Parenthood “Birth Control Quiz”](https://tools.plannedparenthood.org/bc/birth_control_quiz) | Online questionnaire that provides 3 recommended methods based on user’s indicated values | Unknown | Unknown | None | **No.** Unable to access summary of all CM options within the survey. Provides video summaries of your recommended methods only | **No.** For each recommended method, provides 1-3 stars for each user indicated value, but does not include other characteristics or other method options | **Yes.** Explicitly asks user to indicate their values | **No.** Provides resources for user to find a healthcare provider, but no resources for discussion |
| [My Birth Control](https://clinic.mybirthcontrol.org/) | Tablet-based decision support tool for use prior to contraceptive counseling visit | Women aged 15-45 | Yes | [Dehlendorf 2019](https://www.sciencedirect.com/science/article/abs/pii/S0002937819303564?via%3Dihub), Cluster randomized trial, clinical setting (N=749 women ages 15-45) | **Yes.** Provides information about CM options and provides recommendations based on user preferences | **Yes.** Provides information about all CM options and allows users to compare methods | **Yes.** Explicitly elicits patient preferences regarding CM characteristics | **Yes.** Provides a final profile of patient information that can guide the discussion with the provider |
| [Hello Options](https://www.hellogreenlightpro.org/hello-options-tool) | Physical ring with 10 CM options, color-coded by method type (ex. LARC, combined-hormonal, barrier) | AYA aged 12-29 | Yes | [Young Lee 2021](https://www.sciencedirect.com/science/article/abs/pii/S1083318820303478?via%3Dihub), pilot evaluation, clinical setting (N=33 female, 4 women, 2 cisgender female, 1 nonbinary ages 15-29) | **No.** Tool requires a healthcare provider to provide details regarding method use. | **No.** Tool requires a healthcare provider to provide details regarding method effectiveness, side effects, and other characteristics | **No.** Tool requires a healthcare provider to clarify patient values. | **No.** Tool does not provide guidance for discussion |
| [Health-E You/Salud iTu](https://healtheyouinfo.ucsf.edu/) | Mobile application to be used prior to clinic visit to support contraceptive method decision making by eliciting user values, preferences and providing education | Adolescents, with focus on Latina Adolescents | Yes | [Tebb 2017](https://bmjopen.bmj.com/content/8/1/e018201), [2019](https://mhealth.jmir.org/2019/3/e11163/), [2021](https://www.sciencedirect.com/science/article/pii/S0010782421000597?via%3Dihub), cluster RCT, clinical setting, (N=1,360 Latinas ages 14-18) | **Yes.** Provides users with recommended CMs based on their responses, with information about each recommended method | **Yes.** Allows user to learn about all CM options in addition to their recommended methods | **Yes.** Elicits patient preferences and values | **Yes.** Provides guidance for conversation with provider and provides patient responses to provider |
| PreCounselor | Mobile application with comprehensive information about CM options to be used prior to clinic visit | Women aged 15-30 years, with focus on African-American | Unclear if testers (n=17) were under age 18* | [Gilliam 2014](https://www.sciencedirect.com/science/article/pii/S000293781400502X?via%3Dihub), RCT, clinical setting (N=52 women ages 16-30) | **Yes.** Provides information about CM options | **Somewhat.** Informs users about effectiveness of methods and other characteristics, but emphasizes method effectiveness | **No.** Does not explicitly elicit patient values | **No.** App does not provide guidance for visit with provider |
| miPlan iOS application | Refined version of “PreCounselor” with optimized content and updated videos | Women aged 15-29 years, with focus on African-American and Latina women | No (testers 18 and older) | [Hebert 2018](https://www.sciencedirect.com/science/article/abs/pii/S0010782418302403?via%3Dihub), RCT, clinical setting (N=207 women ages 15-29) | **Yes.** Provides information about CM options | **Somewhat.** Only includes videos for LARC methods | **No.** Does not explicitly elicit patient values | **No.** App does not provide guidance for visit with provider |

Table S1: Comparative summary of available contraceptive decision aids.

AYA = Adolescents and young adults, includes people ages 13-25 per the [Society for Adolescent Health and Medicine](https://www.sciencedirect.com/science/article/pii/S1054139X17301647?via%3Dihub).

CM = Contraceptive Method

RCT= Randomized Controlled Trial

SOGIE = Sexual Orientation, Gender Identity or Expression
