# Supplementary material for: Development of a User-Informed Decision Aid for Contraceptive Decision-Making Among Adolescents and Young Adults: Qualitative Study
Source: JMIR Form Res. 2026 Jul 21;10:e82306. doi: 10.2196/82306 (PMC13439044; doi:10.2196/82306)
Supplement: Supplementary file 2 [file formative_v10i1e82306_app5.docx]

| DA^a^ section | Content description | Stakeholder exemplar quotes | Changes implemented based on stakeholder user testing^b^ |
| --- | --- | --- | --- |
| Introduction screen | Welcomes users to the DA, describes its purpose, and explains data protection and confidentiality | - “It’s just like the first sentence is like, [inaudible] make decisions about birth control to prevent unwanted pregnancy, but people use birth control for various means. I just feel like the first sentence alienates people.” [AYA^c^, round 1] | - Explicitly stated that users’ responses will be available to their health care provider - Removed the emphasis on preventing “unplanned pregnancy” from the introduction page |
| User profile | Asks users about their demographic characteristics, gender identity, and sexual orientation | - “They’re missing anything that includes intersex people at all.” [AYA, round1] - “I think they pretty inclusive, especially the gender identity question. I feel like I haven’t seen many apps that have these many options.” [AYA, round 2] | - Added an intersex option - Increased gender identity options and added multiple options selection - Incorporated questions that pertain to the surgical removal of reproductive organs - Removed demographics questions unrelated to CM^d^ recommendation algorithm (eg, education level) |
| User contraceptive need selection | Uses a value clarification method to elicit users’ primary reason for seeking a CM (eg, menstrual suppression or pregnancy prevention) | - “As is, I would explain it primarily as a pregnancy prevention method control app. I think it has the potential to be more than that.” [AYA, round 1] | - Elicit users’ motivations early in the DA to begin DA in a goal-directed, patient-centered manner |
| User contraceptive preferences | Uses value clarification method to elicit user preferences related to CM attribute (eg, remembering something daily, effects on menses, and need to hide CM from partner or family) | - “I would’ve liked to see some questions about menses suppression desires. None of that was actually in there.” [AYA, round 1] - “I like that menstrual suppression is an explicit option for the use of birth control. I think it is not always considered in other questionnaires and things that I’ve taken about reproductive health. It’s always, are you looking to prevent a pregnancy?” [AYA, round 2] - “I think overall, I just felt like this app gives—is very respectful to the patient. I think that just it… gives them agency. It reminds them or allows them to know that they have a choice.” [Provider, round 2] | - User can identify preferred CM attributes using a sliding scale - CM benefits include a range of benefits beyond pregnancy prevention |
| Reproductive health history | Menstrual history, emergency contraception screening, CM use history, pregnancy history, and future family planning (eg, birth spacing) | - “It said male versus female condoms, and I think using the word internal versus external would be a better switch for that because not all males have penises, not all females have vaginas and vice versa.” [AYA, round 1] | - Removed vague language (eg, “sex without a condom” rather than “unprotected sex”)Removed gendered language (eg, “external condom” rather than “male condom”) |
| Health history | Pertinent health history questions informed by the CDC^e^ US Medical Eligibility Criteria | - “I like that the almost exclusively, scientifically correct names were used for things. I think that both eliminated a lot of ambiguity, but also honestly makes things more inclusive because you were just calling it what it is, you’re not assigning a gender or a sexual identity to it. And I really appreciate that.” [AYA, round 2] | - Incorporated health history into CM recommendation algorithm - Expanded health history to include CDC US Medical Eligibility Criteria for Contraceptive Use and cardiometabolic risk and integrated into decision algorithm [13] - Updated wording to fifth-grade reading level |
| CM recommendations page | Provides a CM summary based on CM preference and health history and includes an additional navigation button that displays all CMs | - “I think it’s great because it provides a nice level of understanding and information from which to develop questions and things you want to know more about while making this kind of a decision. So I do think it’s really helpful, especially that it’s so focused on being inclusive of communities that are often marginalized from reproduction healthcare; so I think it’s really helpful.” [AYA, round 2] | - Created “pros” and “cons” columns that incorporate pregnancy prevention and noncontraceptive benefits statistics (eg, menstrual suppression) to decrease emphasis on pregnancy prevention as the “most important” CM indication - Changed illustration to increase diverse representation (race and body size) |
| Provider dashboard | Summarizes the user’s DA responses and CM recommendations and is accessible to the provider before the patient visit | - “I would love to get this information. I mean, it sounds very timesaving just to get an initial, you know, survey of what the patient thinks.” Provider, round 2] | - N/A ^f^— |

^a^DA: decision aid.

^b^The list of implemented changes is not exhaustive but reflects emerging themes from the consensus coding analysis.

^c^AYA: adolescent and young adult.

^d^CM: contraceptive method.

^e^CDC: Centers for Disease Control and Prevention.

^f^ N/A: Not applicable. Provider suggestions for implementing the decision aid in clinical practice were beyond the scope of the decision aid’s development phase and will be evaluated in subsequent pilot and feasibility trials.
